# Disability, social functioning and school inclusion among older children and adolescents living with HIV in Zimbabwe

**DOI:** 10.1111/tmi.13012

**Published:** 2017-12-26

**Authors:** Ruramayi Rukuni, Grace McHugh, Edith Majonga, Katharina Kranzer, Hilda Mujuru, Shungu Munyati, Kusum Nathoo, Celia L. Gregson, Hannah Kuper, Rashida A. Ferrand

**Affiliations:** ^1^ Biomedical Research and Training Institute Harare Zimbabwe; ^2^ Nuffield Department of Population Health University of Oxford Oxford UK; ^3^ Clinical Research Department Faculty of Infectious and Tropical Diseases London School of Hygiene and Tropical Medicine London UK; ^4^ Department of Paediatrics University of Zimbabwe Harare Zimbabwe; ^5^ The Musculoskeletal Research Unit School of Clinical Sciences University of Bristol Bristol UK; ^6^ International Centre for Evidence in Disability London School of Hygiene and Tropical Medicine London UK

**Keywords:** disability, adolescents, children, Africa, HIV, social functioning, invalidité, adolescents, enfants, Afrique, VIH, fonctionnement social

## Abstract

**Objective:**

Increasing numbers of children with HIV are surviving to adolescence and encountering multiple clinical and social consequences of long‐standing HIV infection. We aimed to investigate the association between HIV and disability, social functioning and school inclusion among 6‐ to 16‐year‐olds in Zimbabwe.

**Methods:**

HIV‐infected children receiving antiretroviral therapy from a public‐sector HIV clinic and HIV‐uninfected children attending primary care clinics in the same catchment area were recruited. Standardised questionnaires were used to collect socio‐demographic, social functioning and disability data. Multivariable logistic regression was used to assess the relationship between HIV status and disability and functioning.

**Results:**

We recruited 202 HIV‐infected and 285 HIV‐uninfected children. There was no difference in age and gender between the two groups, but a higher proportion of HIV‐infected children were orphaned. The prevalence of any disability was higher in HIV‐infected than uninfected children (37.6% *vs*. 18.5%, *P* < 0.001). HIV‐infected children were more likely to report anxiety (adjusted odds ratio (aOR) 4.4; 95% CI 2.4, 8.1), low mood (aOR 4.2; 2.1, 8.4) and difficulty forming friendships (aOR 14.8; 1.9, 116.6) than uninfected children. Children with HIV also reported more missed school days, repeating a school year and social exclusion in class. These associations remained apparent when comparing children with HIV and disability to those with HIV but no disabilities.

**Conclusions:**

Children with HIV commonly experience disabilities, and this is associated with social and educational exclusion. Rehabilitation and support services are needed to facilitate educational attainment and social participation in this group.

## Abstract (fr)

**Objectif:**

Un nombre croissant d'enfants infectés par le VIH survivent jusqu’à l'adolescence et sont confrontés aux multiples conséquences cliniques et sociales d'une infection à VIH de longue durée. Nous avons cherché à étudier l'association entre le VIH et l'invalidité, le fonctionnement social et l'inclusion scolaire chez les personnes de 6 à 16 ans au Zimbabwe.

**Méthodes:**

Des enfants infectés par le VIH recevant un traitement antirétroviral dans une clinique VIH du secteur public et des enfants non infectés par le VIH fréquentant des cliniques de soins primaires dans la même zone de recrutement ont été recrutés. Des questionnaires standardisés ont été utilisés pour recueillir des données sociodémographiques, de fonctionnement social et d'invalidité. La régression logistique multivariée a été utilisée pour évaluer la relation entre le statut VIH et l'invalidité et le fonctionnement.

**Résultats:**

Nous avons recruté 202 enfants infectés et 285 enfants non infectés par le VIH. Il n'y avait pas de différence d’âge ni de sexe entre les deux groupes, mais une proportion plus élevée des enfants infectés par le VIH étaient orphelins. La prévalence de toute invalidité était plus élevée chez les enfants infectés par le VIH que chez ceux non infectés (37,6% contre 18,5%; p <0,001). Les enfants infectés par le VIH étaient plus susceptibles de rapporter de l'anxiété (rapport de cotes ajusté [ORa]: 4,4; IC95%: 2,4 ‐ 8,1), un état dépressif (ORa: 4,2; IC95%: 2,1 ‐ 8,4) et des difficultés à nouer des amitiés (ORa: 14,8; IC95%: 1,9 ‐ 116,6) que les enfants non infectés. Les enfants infectés par le VIH ont également rapporté plus de journées d’école manquées, la répétition d'une année scolaire et l'exclusion sociale en classe. Ces associations sont restées apparentes lorsque l'on compare des enfants ayant le VIH et une invalidité à ceux ayant le VIH mais pas d'invalidité.

**Conclusions:**

Les enfants infectés par le VIH souffrent souvent d'invalidités, ce qui est associé à l'exclusion sociale et éducative. Des services de réadaptation et de soutien sont nécessaires pour faciliter l'accès à l’éducation et la participation sociale chez ce groupe.

## Introduction

Of the estimated 1.8 million children who are infected with HIV, 90% live in sub‐Saharan Africa (SSA) 1. The dramatic improvement in survival that has resulted from the global scale‐up of antiretroviral therapy (ART) programmes has meant that increasing numbers of children with HIV who would have died without treatment in infancy are now surviving to older childhood and adolescence. There is increasing evidence that childhood HIV infection is associated with chronic multi‐system complications, resulting in hearing, cognitive, mobility and visual impairments 2, 3.

HIV may lead to impairments through sequelae of infections that occur as a result of immunosuppression. For example, HIV‐mediated immunosuppression leads to opportunistic infections such as CMV that can result in visual impairment 4. The risk of impairments is increased if initiation of ART is delayed, as is common in many resource‐limited settings 5. ART itself may also contribute to impairment; for instance, nucleoside analogue reverse‐transcriptase inhibitors (NRTI) commonly used at the time of ART roll‐out for children in sub‐Saharan Africa (SSA) (e.g. stavudine and lamuvidine) have been linked to hearing loss 6, 7. Zidovudine has been independently linked to myopathy 8, which may lead to physical disability. Once established, impairments may not necessarily be completely reversed by ART 9.

These impairments may, in turn, negatively impact on social functioning and schooling 3, 10. In other words, HIV or its treatment may lead to disability, which is conceptualised as an individuals’ inability to ‘full and effective participation in society on an equal basis of others’ because they have an underlying impairment and face attitudinal and environmental barriers 11. Social deprivation, often associated with HIV infection 12, could potentially exacerbate disability by further restricting participation in society. To optimise the quality of life and long‐term care among those living with HIV and their families, HIV programmes will need to broaden their focus and address longer‐term consequences of HIV infection, including the impact on schooling and social inclusion. Even in the absence of HIV, education and schooling are a major global concern for children and adolescents with disabilities, who are substantially less likely to be enrolled in school and, even when enrolled, lag behind their peers in educational attainment 13. HIV is likely to magnify these issues among children.

We conducted a cross‐sectional study to investigate the association between HIV and disability, social functioning and school inclusion among HIV‐infected children compared to uninfected peers in Zimbabwe.

## Methods

### Study setting and participants

HIV‐infected children aged 6 to 16 years and receiving either first or second line ART for at least 6 months were consecutively recruited from Harare Central Hospital (HCH); this is the largest public‐sector hospital in Harare, providing HIV care for more than 3000 children. Acutely ill children, not residing in Harare and without guardian consent and/or participant assent, were excluded. Recruitment was restricted to the first five eligible participants a day for logistical ease.

A comparison group of HIV‐uninfected children aged 6–16 years were recruited from primary health care clinics (PHC) in seven high‐density communities in the same catchment area served by the clinic from which the HIV‐infected participants were enrolled. Provider‐initiated HIV testing and counselling are offered by PHCs to all attendees regardless of the reason for presentation.

### Data collection

Socio‐demographic data including age, sex and orphan status were recorded. Trained research nurses administered standardised questionnaires to collect data on disability, education and social functioning. The Washington Group/UNICEF Child Functioning and Disability 21‐Question Set was administered to children and their caregivers to assess disability 14. This question set is validated for children aged 2–17 years. Difficulty functioning was defined as binary variables based on self‐reported difficulty in the following domains: vision, hearing, walking, speech, learning, memory, self‐care, anxiety, low mood, difficulty controlling behaviour, dealing with change, forming friendships and concentration. Disability was defined as reported difficulties in any of the functional domains. Additional information on school and social functioning was also collected including school enrolment, school attendance, repeated school grade, problems getting help from teachers and friends, interaction with other children (leadership, play, bullying) and inclusion in lessons and school activities. Caregivers of HIV‐infected children were asked additional questions relating to HIV diagnosis, testing, ART history and children's awareness of diagnosis. CD4 count was determined using an Alere PIMA CD4^+^ (Waltham, Massachusetts, USA), and HIV viral load was measured using COBAS Ampliprep/Taqman 48 Version 2.0 (Roche, Rotkreuz, Switzerland).

### Ethics

Ethical approval was obtained from the Medical Research Council of Zimbabwe (MRCZ/A/1856), the Biomedical Research and Training Institute (AP125), Institutional Review Board, Harare Hospital Ethics Committee and the London School of Hygiene and Tropical Medicine (LSHTM) Ethics Committee (8263). All guardians gave written consent, and participants gave assent to participate in the study.

### Data management and analysis

Data were collected using paper forms and entered into a Microsoft Access (MSACCESS) database using optical mark recognition software (Cardiff TELEFORM Intelligent Character, Version 10.7). Paper forms were manually checked for missing data and inconsistencies before being captured using TELEFORM which has inbuilt quality control checks. Further internal and external consistency checks were carried out after the data were committed into the database using MSACCESS database queries.

Data completeness was assessed by summary and descriptive statistics. There was a low proportion of missing data (<6%) in HIV‐infected and negative children for demographic, clinical, disability and school functioning and inclusion data. All statistical analyses were carried out using Stata v13.0 (College Station, Texas: StataCorp LP). The prevalence of functional difficulties and disability by HIV status was summarised as frequencies and percentages for each variable. Continuous variables were summarised as mean (SD) when normally distributed and median (IQR) when not. Univariable logistic regression analysis was used to compare functional, school and social outcomes between HIV‐infected and uninfected children. Multivariable logistic regression was used to adjust each functional outcome of interest for *a priori* defined variables of age and sex. Orphan status and previous infection/co‐morbidity did not significantly affect the fit of the model (*P* < 0.05) on likelihood ratio testing and therefore were excluded. Hence, the final model was adjusted for age and sex alone.

## Results

### Baseline characteristics of participants

We recruited 202 HIV‐infected children (median age 11 years [IQR 8–13]; 48.8% female) and 285 uninfected children (median age 10 years [IQR 8–13]; 48.8% female). There was no significant difference in age or sex between the two groups, but HIV‐infected children were more likely to be orphaned (aOR 5.2; 3.5, 7.8) (Table 1). Among HIV‐infected children, the median age at HIV diagnosis was 5 years [IQR 3–7] and the median duration of ART was 2 years [IQR 0–5]. The median CD4 count was 726 cells/μl [IQR 476–941].

**Table 1:** Baseline characteristics of HIV‐infected and HIV‐uninfected children in Zimbabwe

Characteristic	HIV+ *n* = 202 *n* (%)	HIV− *n* = 285 *n* (%)	*P* value
Age			
Median (IQR) years	11 (8–13)	10 (8–13)	0.61^a^
6–11 years	132 (65.4)	165 (57.9)	0.06^b^
12–16 years	70 (34.6)	32 (42.1)	
Sex			
Female	97 (48.0)	139 (48.8)	0.11^b^
Orphan status			
Single orphan	69 (34.2)	25 (8.8)	<0.001^b^
Double orphan	28 (13.9)	7 (2.5)	
Not orphaned	98 (48.5)	245 (85.9)	
Age at HIV diagnosis			
Median (IQR) years	5 (3–7)		
ART duration			
<1 year	75 (37.1)		
1–5 years	97 (48.0)		
>5 years	30 (14.9)		
CD4			
Median (IQR) cells/μl	726 (476–941)		
<200 cells/μl	9 (4.5)		
200–500 cells/μl	47 (23.2)		
>500 cells/μl	144 (71.3)		
Viral load			
Median (IQR) copies/ml	19 (19–250)		
<400 copies/ml	152 (75.2)		
400–5000 copies/ml	14 (7.0)		
>5000 copies/ml	32 (15.8)		

HIV+, HIV‐infected; HIV−, HIV‐uninfected, SD standard deviation.

a *P* value from Mann–Whitney *U* test.

b *P* value from chi‐squared test.

### Functioning and disability

The prevalence of any self‐reported difficulties in functioning (i.e. disability) was higher in HIV‐infected than uninfected children (37.6% compared to 18.5% *P* < 0.001; Table 2). The most common type of disability was problems with memory and learning difficulties, reported by 17.8% and 23%, respectively. Difficulties with seeing (7.7%), hearing (4.8%) and walking (2.5%) were less frequent.

**Table 2:** Domains of disability and functioning in HIV‐infected and HIV‐uninfected children in Zimbabwe

Outcome	HIV+ *n* = 202 *n* (%)	HIV− *n* = 285 *n* (%)	Crude OR (95% CI)	*P* value^a^	aOR (95% CI)	*P* value^a^
Any disability	76 (37.6)	53 (18.8)	2.3 (1.6, 5.3)	<0.001	2.8 (1.8, 4.2)	<0.001
Seeing	16 (7.7)	9 (3.1)	2.7 (1.2, 6.0)	0.009	3.0 (1.3, 6.9)	0.009
Hearing	10 (4.8)	4 (1.4)	3.4 (1.1, 10.6)	0.031	3.4 (1.0, 10.5)	0.036
Walking	5 (2.5)	1 (0.4)	7.4 (0.9, 63.5)	0.065	7.4 (0.9, 63.5)	0.055
Speaking	9 (4.3)	3 (1.1)	4.0 (1.1, 14.5)	0.042	3.8 (1.1, 13.9)	0.042
Learning	48 (23.2)	33 (11.6)	2.1 (1.3, 3.2)	0.002	3.9 (1.4, 3.4)	0.001
Remembering	37 (17.8)	16 (5.6)	3.6 (2.0, 6.6)	<0.001	3.5 (2.0, 6.6)	<0.001
Self‐caring	3 (1.5)	1 (0.4)	1.7 (0.4, 8.0)	0.072	1.6 (0.4, 7.8)	0.524
Anxiety	42 (20.3)	14 (5.6)	4.6 (2.4, 8.2)	0.000	4.4 (2.4, 8.1)	<0.001
Depression	32 (15.5)	12 (4.2)	4.2 (2.1, 8.5)	0.010	4.2 (2.1, 8.4)	0.010
Controlling behaviour	3 (1.5)	1 (0.4)	4.0 (0.4, 39.4)	<0.001	4.0 (0.4, 39.3)	0.003
Concentration	2 (1.0)	6 (2.1)	0.4 (0.1, 2.2)	0.478	0.4 (0.1, 2.2)	0.311
Accepting change	39 (10.9)	36 (12.6)	1.6 (0.9, 2.6)	0.085	1.5 (1.0, 2.5)	0.075
Making friends	10 (4.8)	1 (0.4)	14.6 (1.9, 115.2)	0.001	14.8 (1.9, 116.6)	0.011

HIV+, HIV‐infected; HIV−, HIV‐uninfected; OR, odds ratio; aOR age, sex‐adjusted odds ratio.

a *P* value from chi‐squared test.

After adjustment for age and sex, the odds of any disability were 2.8 times higher in HIV‐infected than HIV‐uninfected children (95% CI 1.8, 4.2 *P* < 0.001). HIV‐infected children were significantly more likely to report visual (aOR 3.0; 1.3, 6.9), hearing (aOR 3.4; 1.0, 10.5), speech (aOR 3.8; 1.1, 13.9), learning (aOR 3.9; 1.4, 3.4) and memory problems (aOR 3.5; 2.0, 6.6; Table 2).

In addition, HIV‐infected children were more likely to report anxiety (aOR 4.4; 2.4, 8.1), low mood (aOR 4.2; 2.1, 8.4) and difficulty forming friendships (aOR 14.8; 1.9, 116.6) than their uninfected peers. There was no significant association between age at HIV diagnosis, CD4 count, viral load, ART duration or previous co‐morbidity and disability among HIV‐infected children (Table 3).

**Table 3:** Difference in HIV characteristics among HIV‐infected children with and without disability in Zimbabwe

Characteristic	HIV+ with disability: *n* = 76	HIV+ without disability: *n* = 126	*P* value	
Age				
Mean (SD) years	10.9 (2.6)	10.3 (2.6)		
6–9 years	24 (31.6)	48 (38.1)	0.77	‐
10–12 years	31 (40.8)	50 (39.7)		
13–14 years	15 (19.7)	20 (15.9)		
15–16 years	6 (7.9)	8 (6.4)		
Age at diagnosis				
Mean (SD) years	5.0 (3.0)	5.1 (2.9)		‐
Sex				
Female	35 (46.0)	62 (49.2)	0.66	
				aOR (95% CI)
CD4 count				
Median (IQR) cells/μl	736 (513–914)	720 (459–910)		
<200 cells/μl	3 (4.0)	6 (4.7)	0.78	1.0
200–500 cells/μl	15 (19.7)	32 (25.4)		1.4 (0.8, 2.5)
>500 cells/μl	57 (75.0)	87 (69.1)		
Viral load				
Median (IQR) copies/ml	19 (19–190)	19 (19–343)		
<400 copies/ml	57 (75.0)	95 (75.4)	0.16	1.0
400–5000 copies/ml	2 (2.6)	12 (9.5)		1.1 (0.7, 1.6)
>5000 copies/ml	14 (18.4)	18 (14.3)		
ART duration				
Median (IQR) years	2 (0–5)	1 (0–4)		
<1 year	24 (31.6)	51 (40.5)	0.21	1.0
1–5 years	39 (51.3)	58 (46.0)		(0.8, 1.9)
>5 years	13 (17.1)	17 (13.5)		
No of hospital admissions in 12 months				
>1	5 (6.6)	5 (4.0)		1.9 (0.6, 6.1)
Past history of TB	29 (38.2)	50 (39.7)	0.94	0.9 (0.5, 1.6)

HIV+, HIV‐infected; HIV−, HIV uninfected; aOR, odds ratio adjusted for age and sex; ART, antiretroviral therapy; TB, tuberculosis.

### Schooling and social inclusion

School enrolment rates were high among all children (96.0% in both HIV‐infected and uninfected groups). However, children living with HIV were more likely to have repeated a school year (aOR 3.2; 1.6, 3.8) and on average missed more days of school in the preceding month (mean 0.9 days (range 0–15 days) *vs*. 0.3 days (range 0–7 days). HIV‐infected children more frequently reported not getting help from teachers (aOR 2.1; 1.2, 3.8) or friends (aOR 3.0; 2.0, 4.5) at school. They were more likely to feel excluded in lessons and activities (aOR 4.7; 2.7, 8.3) and more likely to be hit, hurt or have nasty things said to them by other children (aOR 3.7; 2.2, 6.0). Among children with HIV, those with disabilities were less likely to be enrolled in the same school grade as their peers (aOR 3.3; 1.7, 6.1) and more likely to repeat a school year (aOR 1.9; 1.0, 3.6) than HIV‐infected peers without disability. They were also more likely to report that their peers did not look up to them as leaders (aOR 2.1; 1.4, 3.4) and that they experienced violence from their peers (aOR 2.5; 1.3, 4.8) (Table 4).

**Table 4:** School and social inclusion at school in HIV‐infected and HIV‐uninfected children and in HIV‐infected children with and without disability

Characteristic	HIV+ *n* = 202 *n* (%)	HIV− *n* = 285 *n* (%)	aOR (95% CI)	HIV+ with disability *n* = 76 *n* (%)	HIV+ without disability *n* = 126 *n* (%)	aOR (95% CI)
School inclusion as reported by children and their carers						
Currently enrolled in school	194 (96.0)	273 (96.0)	0.98 (0.4, 2.5)	71 (93.4)	123 (97.6)	0.3 (0.1, 1.5)
Enrolled in the same grade as peers	102 (50.5)	197 (69.1)	2.4 (1.6, 3.6)	24 (31.6)	78 (61.9)	3.3 (1.7, 6.1)
Ever repeated a year at school	68 (33.7)	53 (18.6)	2.5 (1.6, 3.8)	32 (42.1)	36 (28.6)	1.9 (1.0, 3.6)
Social inclusion at school as reported by children and their carers						
No help from teachers, if problem at school	4 (7.4)	2 (0.7)	2.1 (1.2, 3.8)	2 (2.6)	2 (1.6)	1.7 (0.9, 3.2)
No help from friends, if problem at school	15 (7.4)	3 (1.1)	3.0 (2.0, 4.5)	11 (14.5)	4 (3.2)	1.5 (0.9, 2.4)
Child has no friends to play with	2 (1.0)	1 (0.4)	1.8 (0.7, 5.0)	2 (2.6)	1 (0.8)	1.7 (0.8, 5.7)
Friends look up to child as a leader	108 (53.5)	147 (51.6)	1.1 (0.8, 1.6)	44 (57.9)	41 (32.5)	2.1 (1.4, 3.4)
Other children hit, hurt /say nasty things to child	58 (28.7)	28 (9.8)	3.7 (2.2, 6.0)	30 (39.5)	28 (22.2)	2.5 (1.3, 4.8)
Child does not feel included in lessons and activities	6 (3.0)	2 (0.7)	4.7 (2.7, 8.3)	3 (4.0)	3 (2.4)	0.6 (0.1, 3.0)

HIV+, HIV‐infected; HIV−, HIV Uninfected; aOR, odds ratio adjusted for age and sex.

## Discussion

This study demonstrates a high prevalence of physical and cognitive functional difficulties in HIV‐infected children. HIV‐infected children are more likely to report low mood/anxiety and difficulty forming friendships, repeating a school year and to experience poor social support at school, particularly those who have both HIV and disability.

Other studies have reported increased physical, sensory and cognitive difficulties in HIV‐infected children compared to uninfected children 3, 15, 16, 17, 18, 19, 20, 21. In SSA, developmental delay (global, motor and cognitive) is strongly associated with HIV 2, with prevalence as high as 78% in some children 21. A number of studies assessed the prevalence of motor impairment 17, 18, 20, 22, 23, 24, 25, 26, 27 and cognitive impairment among HIV‐infected and uninfected children 17, 21, 23, 25, 26, 27. However, studies to date have largely focused on infants and younger children using differing neurodevelopment assessment measures, thereby making it difficult to compare findings. Although studies in high‐income countries have also found a detrimental effect of childhood HIV on neurocognitive development, they have similar limitations 28, 29, 30. Severe forms of cognitive impairment in children appear to be decreasing in the post‐ART era in high‐income countries, but the prevalence of mild impairment is largely unchanged and may be increasing 31.

Our study showed that HIV has a negative association with forming friendships, school progression and inclusion in SSA, and this is worse among children with disability. A recent cross‐sectional study of hearing impairment in 380 HIV‐infected Malawian children with extensive audiological testing (otoscopy, tympanometry, transient evoked oto‐acoustic emissions and audiometry) showed that one quarter (24%) of children had hearing loss, and of those with hearing loss, 23% required a hearing aid to be fitted. Children with hearing impairment were less likely to attend school and had poorer emotional and school functioning than HIV‐positive children without hearing loss. Furthermore, only 40% of caregivers accurately perceived their child's hearing loss, and few had sought treatment, implying that routine screening may be necessary 10.

Evidence in the wider literature for the link between disability and HIV disease severity and treatment factors has been inconsistent. Previous studies have shown an association between CD4, viral load at enrolment, ART duration and disability. The above‐mentioned study in Malawi 10 showed that hearing loss was significantly associated with history of frequent ear infections and ear drainage, malnutrition, history of HIV, WHO Stage 3 or 4 disease, but not duration of ART or CD4. A recent systematic review of studies in SSA showed that fifteen of thirty‐one (48%) studies found a statistically significant dose–response relationship between indicators of disease progression (CD4 or WHO stage) and disability 2. Earlier treatment of children may reduce the risk of impairments and consequent disability, but once established, ART alone will be insufficient to enable children with HIV to lead healthy lives.

One study reviewed the inclusion of disability within 18 national strategic responses to HIV and AIDS in Eastern and Southern Africa and identified that 9 of 18 countries (50%) provided HIV prevention and only 5 of 18 countries (27%) recognised the need for specific impact mitigation and support services interventions for people with disabilities 32. Incorporating disability‐inclusive approaches to HIV prevention, treatment and care may increase the social participation and school functioning of children with HIV.

To our knowledge, this is the first study to estimate the prevalence of disability and its association with school and social functioning in HIV‐infected children. A comparison group was included, and participants were not selected on the basis of symptoms. Limitations of this study include possible misclassification and/or recall bias from the use of self‐reported outcome measures for functional difficulties and disability without contemporaneous clinical measures of impairments. There was also no information available on the nature and cause of these impairments. In addition, there is the possibility of selection bias and unknown confounding through the use of non‐probability‐based sampling. Unfortunately, socio‐economic data such as household income and size, asset ownership, caregiver education and food security were not available which meant that analyses could not be adjusted for socio‐economic status.

Although it is evident that disability is common in HIV‐infected children and has a major impact on their lives, further epidemiological and operational research specifically analyses each type of impairment to understand which interventions are required to inform the design of effective rehabilitation services and guide policy decisions. Examples of the type of interventions for HIV‐infected children that could be introduced include (i) routine screening for impairments (ii) linking HIV care to rehabilitation and additional clinical services (e.g. ENT in the case of hearing impairment) (iii) interventions to promote school inclusion and social acceptance among children with HIV (e.g. through training of parents, teachers and peers).

## Conclusion

This study suggests that physical and cognitive functional difficulties are common among children with HIV. These difficulties are associated with school exclusion, including impaired educational progress, difficulty forming friendships and reduced ability to participate in lessons and activities. Further work is required to develop tools to better detect and understand the need for rehabilitation and support services within paediatric HIV programmes.
